# New Insights into the TIFY Gene Family of *Brassica napus* and Its Involvement in the Regulation of Shoot Branching

**DOI:** 10.3390/ijms242317114

**Published:** 2023-12-04

**Authors:** Yarong Li, Qian Zhang, Luman Wang, Xinfa Wang, Jiangwei Qiao, Hanzhong Wang

**Affiliations:** Laboratory of Biology and Genetic Improvement of Oil Crops, Oil Crops Research Institute of the Chines Academy of Agricultural Sciences, Ministry of Agriculture and Rural Affairs, Wuhan 430062, China; yarongli1993@163.com (Y.L.); zq74079@163.com (Q.Z.); wlm990907@163.com (L.W.); wangxinfa@caas.cn (X.W.); wanghz@oilcrops.cn (H.W.)

**Keywords:** *Brassica napus*, TIFY gene family, axillary buds, shoot branching, jasmonic acid

## Abstract

As plant-specific transcription factors, the TIFY family genes are involved in the responses to a series of biotic and abiotic stresses and the regulation of the development of multiple organs. To explore the potential roles of the TIFY gene family in shoot branching, which can shape plant architecture and finally determine seed yield, we conducted comprehensive genome-wide analyses of the TIFY gene family in *Brassica napus*. Here, HMMER search and BLASTp were used to identify the TIFY members. A total of 70 TIFY members were identified and divided into four subfamilies based on the conserved domains and motifs. These TIFY genes were distributed across 19 chromosomes. The predicted subcellular localizations revealed that most TIFY proteins were located in the nucleus. The tissue expression profile analyses indicated that TIFY genes were highly expressed in the stem, flower bud, and silique at the transcriptional level. High-proportioned activation of the dormant axillary buds on stems determined the branch numbers of rapeseed plants. Here, transcriptome analyses were conducted on axillary buds in four sequential developing stages, that is, dormant, temporarily dormant, being activated, and elongating (already activated). Surprisingly, the transcription of the majority of TIFY genes (65 of the 70) significantly decreased on the activation of buds. GO enrichment analysis and hormone treatments indicated that the transcription of TIFY family genes can be strongly induced by jasmonic acid, implying that the TIFY family genes may be involved in the regulation of jasmonic acid-mediated branch development. These results shed light on the roles of TIFY family genes in plant architecture.

## 1. Introduction

The TIFY gene family was originally identified in *Arabidopsis thaliana* (*A. thaliana*) [1]. As plant-specific transcription factors, they were identified to be involved in multiple plant hormone signaling pathways, especially those of jasmonic acid (JA), gibberellin, abscisic acid, auxin, and ethylene [2]. The TIFY gene family is characterized by 36 highly conserved amino acids (TIF[F/Y] XG) within the TIFY domain and is divided into four subfamilies: TIFY, JAZ, PPD, and ZML [2,3,4]. The TIFY subfamily contains only a TIFY motif [3,4]; JAZs is the largest subfamily in the TIFY gene family and possesses two conserved domains: a TIFY domain with the core motif TIF[F/Y] XG at the N-terminus and a jas domain with a unique sequence, SLX2FX2RX2RX5PY, at the C-terminus [4,5,6]. The PPD subfamily contains three domains: an N-terminal PPD domain with about 55 amino acids, a TIFY domain and a jas domain without the proline–tyrosine (PY) amino acid near the C-terminus [3]. The ZML subfamily possesses TIFY, C_2_C_2_-GATA zinc-finger, and CCT domains [7]. In the TIFY domain, glycine is the only one conserved among hydrophobic amino acids [4,8]. The TIFY domain has an α-α-β structure based on secondary structure prediction [9]. The jas domain with the unique characteristic motif of SLX2FX2RX2RX5PY can interact with COI1 (the F-box component of SCF) and other transcription factors, such as GhbHLH and R2R3-MYB transcription factors [10,11,12,13,14,15]. The conserved motifs and specific secondary structures suggest the various functions of TIFY family genes during plant growth and development.

So far, TIFY family genes have been reported to be mainly involved in the responses to biotic and abiotic stresses and the regulation of multiple aspects of plant growth and development [16]. The expression of most *OsTIFYs* is responsive to cold, drought, salt, and heat abiotic stresses in *Fagopyrum tataricum* [17]. Overexpression of *GsTIFY10* in *A. thaliana* enhances the plant’s tolerance to bicarbonate stress [18]. JAZs, as the largest subfamily in the TIFY family, are involved in multiple regulatory pathways. During the growth and development of roots, leaves, and flowers, multiple factors can rapidly activate the expression levels of JAZ genes [19]. The expression of JAZ genes was induced at the early stages of nutrient deficiency followed by a decrease at later stages of macronutrient (N P K) deficiency in rice and chickpea [20]. Osmotic pressure, low temperature, drought and salt stresses, and ABA treatment can induce the expression of *VvJAZ* genes [21]. Abiotic stresses such as drought can also induce a strong response of *ZmJAZ* genes [22]. Furthermore, JAZ proteins play important roles in the JA signal pathway and have been identified as transcriptional inhibitors in the JA pathway [6,23]. In cotton, *GhJAZ1* plays key roles in maintaining the balance between cotton growth and resistance against Verticillium wilt [24]. Overexpression of *GsJAZ2* resulted in enhanced tolerance to salt and alkali stresses in Arabidopsis. Compared with those in wild-type plants, some marker genes of alkali stress responses and stress-inducing responses showed significantly higher expression levels in *GsJAZ2*-overexpression plants [25]. In tobacco, overexpression of *NaJAZd* and *NaJAZh* inhibited the shedding of flower buds by regulating JA levels and promoted the synthesis of nicotine, respectively [26,27]. *AtPPD4* may participate in defense against *geminviruses* [28]. PPD proteins have been related to cell cycle arrest [29]. Overexpression of *ZIM* under the CaMV35S promoter resulted in the elongation of hypocotyls and petioles in Arabidopsis; ZML1 and ZML2 were involved in the cry1-mediated photoprotective responses; MYB/ZML complexes containing ZmZML2 and ZmMYB11 were involved in wound-induced lignification [1,30,31]. In summary, TIFY family genes are important for stress tolerance in plant growth.

In this study, we identified in detail the TIFY family members in the allotetraploid crop *Brassica napus*. The rapeseed TIFY family consists of 70 genes whose tissue expression levels are various, but the functions of most of the TIFY genes are unknown. In order to explore the potential roles of TIFY family genes in shoot branching, we conducted transcriptome analyses of axillary buds in different developing states, which represented the beginning steps of branching. The transcriptional levels of most TIFY family genes in buds were dramatically decreased on the transition from dormancy to activation. GO enrichment analysis and hormone treatments indicated that TIFY family genes strongly responded to JA. These results suggested that TIFY family genes may negatively regulate the development of branching, providing new insights into the functions of TIFY family genes.

## 2. Results

### 2.1. Identification of the TIFY Members and Phylogenetic Analyses of the TIFY Family Genes in Brassica napus

**Figure 1:**
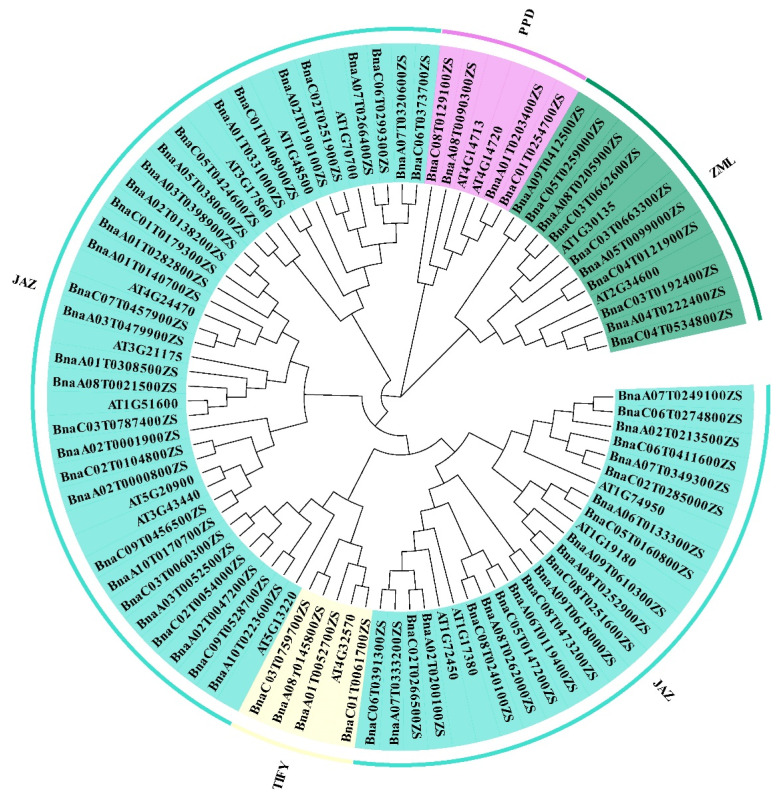
Phylogenetic analysis of the TIFY family genes of *Brassica napus* and *Arabidopsis thaliana*. The protein sequence alignments and construction of the phylogenetic tree were performed using MEGA X and the neighbor-joining method with 1000 bootstrap replicates. The different colors represent four subfamilies of the TIFY gene family; branches indicate different evolutionary clades.

To identify all the TIFY members in rapeseed, we performed BLASTp analyses and HMMER searches in the ZS11.V0 genome (https://yanglab.hzau.edu.cn/BnIR/genome_data, accessed on 3 April 2023) [32] using all amino acid sequences of Arabidopsis TIFY proteins. The results showed that a total of 70 TIFY genes were generated (Table 1). To further understand the evolutionary relationships among the TIFY family members in *Brassica napus*, multiple sequence alignments were performed between TIFY genes in *Brassica napus* and *Arabidopsis thaliana*, and a rootless phylogenetic tree was constructed using MEGA X [33]. As shown in Figure 1, the rapeseed TIFY family is divided into four subfamilies, the JAZ, TIFY, ZIM/ZML, and PPD subfamilies. The JAZ subfamily is the largest subfamily in the TIFY gene family, with 52 genes belonging to the JAZ subfamily. The ZML and TIFY subfamilies each contain 4 genes, and the residual 10 genes belong to the ZML subfamily…

**Figure 2:**
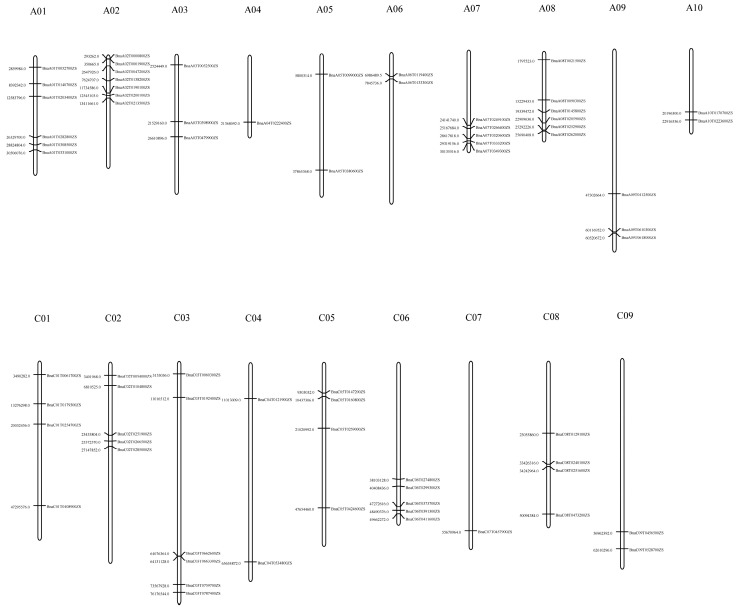
Chromosome locations of rapeseed TIFY family genes. The length of the bars indicates the sizes of *Brassica napus* chromosomes. The physical locations of the genes are labeled on the left of the chromosomes, and the genes are labeled on the right of the chromosomes.

The location analysis showed that rapeseed TIFY genes are distributed across 19 chromosomes, and detailed information on chromosomal location is given in Figure 2. A02 is the chromosome with the largest number of TIFY genes, containing seven genes. Chromosomes A04 and C07 contain only one TIFY gene each. Genes belonging to the JAZ subfamily are located on chromosomes C04, C05, A09, C06, and A07. The ZML subfamily genes are distributed on chromosomes A01, A02, A03, A08, C01, C03, and C07. The TIFY subfamily members are distributed on chromosomes A01, A08, C01, and C03. The PPD subfamily members are distributed on chromosomes A01, A08, C01, and C08. All the rapeseed TIFY genes are distributed across the A and C subgenomes without preference.

The TIFY proteins contain 112 to 367 amino acids. The molecular weight of TIFY proteins ranges from 11.96 KDa to 40.68 KDa and the theoretical isoelectric point (PI) of these proteins ranges from 4.29 to 10.73. The subcellular localization of these proteins predicted by a public website (https://wolfpsort.hgc.jp/, accessed on 12 April 2023) reveals that most (56 of 70) TIFY proteins are located in the nucleus; the remaining 14 proteins are located in the chloroplasts, mitochondria, cytoplasm, and Golgi apparatus. The apparent differences of TIFY proteins in the number of amino acids, isoelectric point, and subcellular localization reveal the evolutionary diversity and functional complexity of TIFY family genes.

### 2.2. Gene Structural Analysis and Motif Composition of the Rapeseed TIFY Family Genes

**Figure 3:**
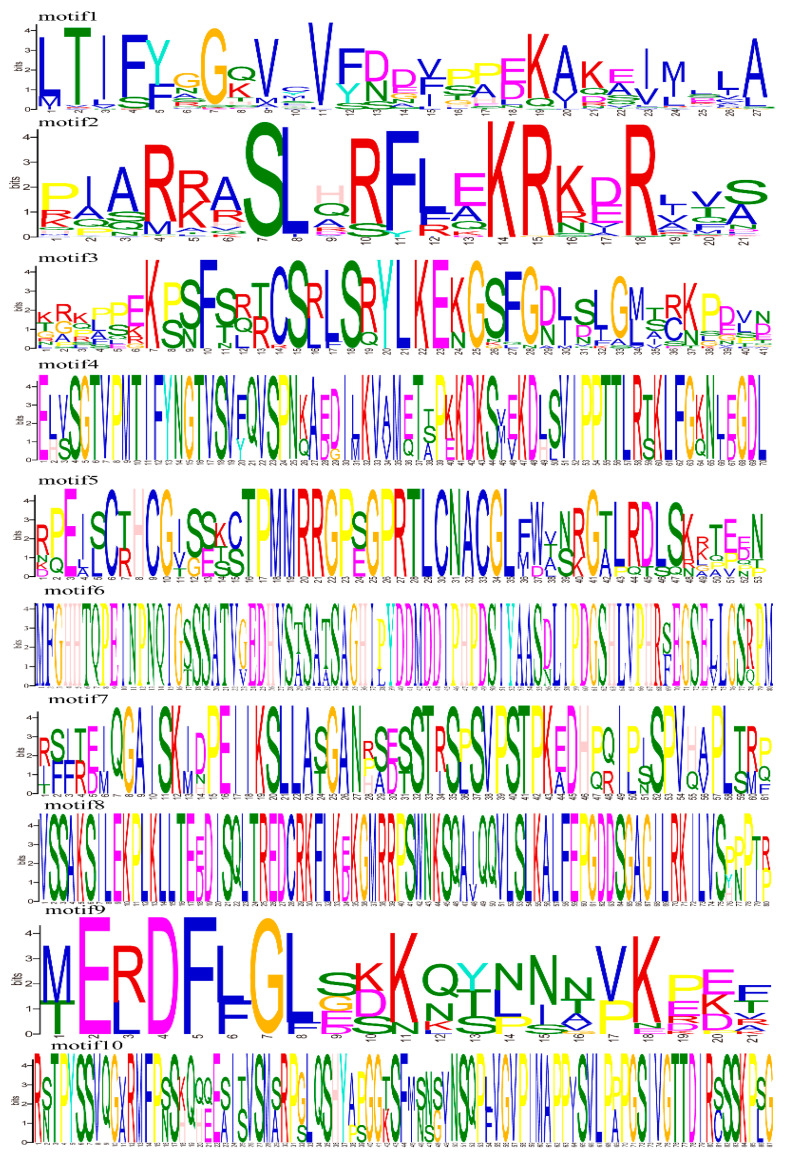
The 10 conserved motifs of rapeseed TIFY genes.

According to the conserved sequences of 70 rapeseed TIFY genes, a total of 10 potential conserved motifs were identified by MEME analysis. The detailed sequences of conserved motifs are shown in Figure 3. Most TIFY family proteins contain 2–4 motifs, except for the proteins of the TIFY subfamily, which contain only one motif. *BnaA01G0331000ZS*, *BnaA05G0380600ZS*, *BnaC01G0408900ZS*, *BnaC05G0424600ZS*, *BnaA02G0047200ZS*, *BnaA03G0052500ZS*, *BnaA10G0223600ZS*, *BnaC02G0054000ZS*, *BnaC03G0060300ZS*, and *BnaC09G0528700ZS* have four motifs. Motif 1 exists in each TIFY protein. Motif 2 exists in all TIFY family proteins except the TIFY subfamily, and motif 4 only exists in *BnaA01G0331000ZS*, *BnaA05G0380600ZS*, *BnaC01G0408900ZS*, and *BnaC05G0424600ZS*. The remaining motifs are distributed in different subfamilies. Therefore, the different functions of TIFY family proteins may be highly correlated with the differences in the number of motifs.

**Figure 4:**
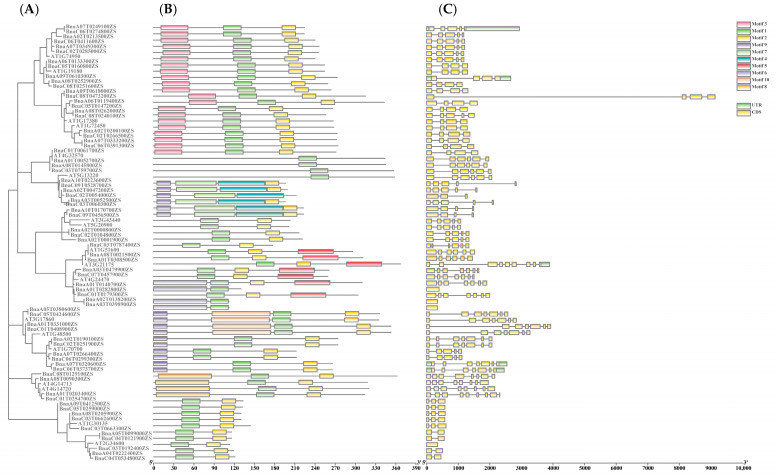
Phylogenetic relationships, gene structures, and conserved motifs of rapeseed TIFY genes. (**A**). The phylogenetic relationships of TIFY family members between rapeseed and Arabidopsis. (**B**). The conserved motif compositions of TIFY family members between rapeseed and Arabidopsis. Different colored boxes in the upper right corner indicate different motifs. (**C**). Gene structure analysis of TIFY family members between rapeseed and Arabidopsis. The yellow boxes indicate exons, and the green boxes indicate UTRs.

In order to understand the structure of TIFY family genes, the exons and introns of each gene were investigated. As shown in Figure 4, the number of exons in TIFY genes ranges from one to nine. The members belonging to one subfamily have similar genetic structures. For example, *BnaA01G0331000ZS*, *BnaA05G0380600ZS*, *BnaC01G0408900ZS*, and *BnaC05G0424600ZS* contain seven exons; TIFY subfamily genes contain six exons; and *BnaA04G0222400ZS*, *BnaA05G0099000ZS*, *BnaC03G0192400ZS*, *BnaC04G0121900ZS*, and *BnaC04G0534800Z* contain three exons. The differences in the number of motifs and exons contribute to the evolutionary diversity of the TIFY family.

### 2.3. The Tissue Expression Profile of TIFY Family Genes in Rapeseed

Zhongshuang11 (ZS11) is an elite and popular semi-winter rapeseed variety. ZS11 has strong ecophysiological adaptation and is also widely popularized in China. In addition, the genome of ZS11 has been sequenced, assembled, and used as the reference genome [34,35,36]. Apart from that, RNA-seq analyses of different tissues covering several key developmental stages in ZS11 have been performed. These data were uploaded to the publicly available database: *Brassica napus* Transcriptome Public Database (https://yanglab.hzau.edu.cn/BnIR/expression_zs11, accessed on 27 April 2023) [37]. A heatmap was drawn based on the public RNA-seq data to understand the expression patterns of TIFY genes in rapeseed. The transcriptional levels of TIFY genes in 13 tissues in different developmental stages were investigated. The tissues include the root, stem (lower stem peel, middle stem peel, upper stem peel), leaf (cotyledon, vegetative rosette leaf; leaf 1, leaf 12, and leaf 23 represent the first, twelfth, and twenty-third leaves on stem from top to bottom), petal, flower bud (4 mm), seed (40 days after flowering), and silique (30 days after flowering), which cover the tissues across the whole rapeseed plant life cycle.

**Figure 5:**
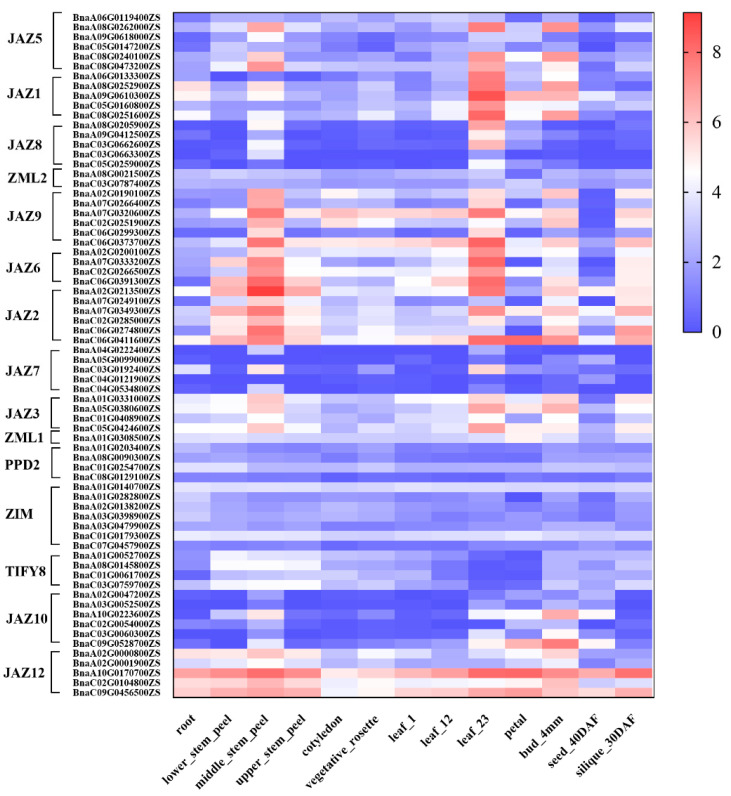
Tissue expression pattern of TIFY family genes. The data are from available public databases [33]. The heatmap was generated using log2 expression levels (TPM + 1). The bar indicates the log2 expression levels (TPM + 1). The detailed gene expression values (TPM) are listed in Table S1.

As shown in Figure 5, most of the rapeseed TIFY genes are highly expressed in the middle stem peel, leaf 23 (representative mature leaf), 4 mm flower bud, and silique of 30 days after flowering. The 18 genes belonging to the ZIM/ZML subfamily, PPD subfamily, and TIFY subfamily are expressed in all tissues at a low level. In different developmental stages, 11 of the 52 JAZ subfamily genes are expressed in tissues at a low level. Sixteen genes, such as *BnaC08G0240100ZS*, *BnaA08G0205900ZS*, and *BnaA09G0610300ZS*, are highly expressed in leaf 23. The 20 genes belonging to the JAZ2, JAZ3, JAZ6, and JAZ9 subfamilies, such as *BnaA07G0349300ZS*, *BnaA01G0331000ZS*, *BnaA07G0333200ZS*, and *BnaA07G0320600ZS*, are highly expressed in the middle stem peel, leaf 23, 4 mm flower bud, and silique of 30 days after flowering. Interestingly, five genes, *BnaA02G0000800ZS*, *BnaA02G0001900ZS*, *BnaA10G0170700ZS*, *BnaC02G0104800ZS*, and *BnaC09G0456500ZS*, are highly expressed in all tissues. Genes of the same subfamily have identical expression patterns in the same tissues. The tissue expression profiles show that TIFY family genes are highly expressed in the stem, flower bud, and silique, suggesting their important functions in rapeseed growth.

### 2.4. The Transcripts of TIFY Family Genes Highly Accumulate in Dormant Axillary Buds and Significantly Decrease in Outgrowing Axillary Buds

**Figure 6:**
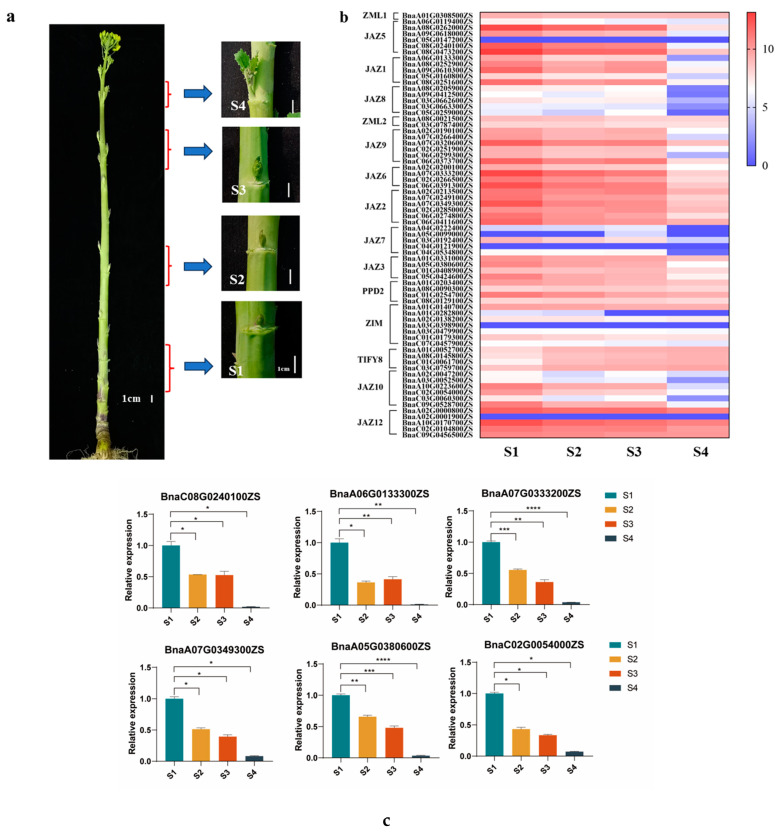
(**a**) Rapeseed axillary buds in different developmental states: dormant (S1), temporarily dormant (S2), being activated (S3), and elongating (S4, already activated). The left shows the bud sampling positions on the main stem (leaves have been removed), and the right indicates enlarged axillary buds in each stage. (**b**) Expression profile of TIFY family genes in different developmental stages of axillary buds. The heatmap was generated using log2 expression levels (Counts + 1). The bar indicates the log2 expression levels (Counts + 1). The expression values (Counts) of all TIFY genes in different axillary buds are listed in Table S2. (**c**) The relative expression of TIFY genes in axillary buds in different states. The expression levels of related genes were calculated with the following equation: 2^−ΔΔCT^. Mean ± standard error is plotted. Significant differences (*p* < 0.05) are indicated by asterisks. * Represents *p* < 0.05, ** represents *p* < 0.01, *** represents *p* < 0.001, **** represents *p* < 0.0001.

Branching is normally divided into two steps: the formation of the axillary buds and their consequent activation and outward growth. One axillary bud exists in each leaf axil of rapeseed plants. With the transition from vegetative growth to reproductive growth, some axillary buds become activated; they then outgrow and elongate and eventually develop into branches. To explore whether TIFY family genes are involved in branch development, the transcriptomic response of TIFY family genes in branching was investigated using whole transcriptome sequencing of axillary buds in four states. The four different states of axillary buds in early branch development were as follows: dormant (S1), temporarily dormant (S2), being activated (S3), and elongating (S4, already activated) (Figure 6a).

To verify the reliability of the transcriptome data, six genes were selected (*BnaC08G0240100ZS*, *BnaA06G0133300ZS*, *BnaA07G0333200ZS*, *BnaA07G0349300ZS*, *BnaA05G0380600ZS*, and *BnaC02G0054000ZS*) for real-time quantitative PCR in axillary buds in different states. As shown in Figure 6c, the highest expression of these genes is shown in dormant axillary buds (S1), and the lowest expression is shown in elongating axillary buds (S4). The expression levels of these genes are slightly lower in the activated axillary buds (S3) than in the temporarily dormant axillary buds (S2). Our results are completely consistent with the transcriptomic results, indicating that these transcriptome data are authentic and reliable for subsequent analysis.

In Figure 6b, only four TIFY family genes (*BnaC05G0147200ZS*, *BnaC04G0121900ZS*, *BnaA03G0398900ZS*, and *BnaA02G0001900ZS*) show no expression in the axillary buds of the four stages. Transcripts of the majority of TIFY family genes highly accumulate in dormant axillary buds (S1) and significantly decrease in axillary buds undergoing elongation (S4). The transcriptional levels of some JAZ subfamily genes in axillary buds are dramatically decreased with the transition from dormancy to activation. Compared with those of dormant axillary buds, the transcriptional levels of *BnaA08G0262000ZS*, *BnaA09G0618000ZS*, and *BnaC08G0251600ZS* in activated axillary buds were downregulated by approximately 33.8, 25.9, and 23.7 times, respectively. The transcriptional levels of *BnaA02G0000800ZS*, *BnaA10G0170700ZS*, *BnaC02G0104800ZS*, and *BnaC09G0456500ZS* are highly expressed in each state of axillary buds without expression fluctuation. *BnaA04G0222400ZS* and *BnaC04G0534800ZS* maintain consistent low transcription in both dormant and activated axillary buds. Interestingly, compared with the dormant axillary buds, the expression of PPD subfamily genes *BnaA01G0203400ZS* and *BnaC01G0254700ZS* decreased by 2.5- and 3-fold in activated axillary buds, respectively. However, the expression levels of *BnaA08G0090300ZS* and *BnaC08G0129100ZS* belonging to the PPD subfamily show no difference among different axillary buds. The transcriptional levels of ZML subfamily genes also show no obvious variation between the dormant and activated axillary buds. To our surprise, TIFY subfamily genes show a low transcriptional level in dormant axillary buds and a high transcriptional level in activated axillary buds, which is an opposite expression pattern. The transcriptional levels of TIFY subfamily genes *BnaA01G0052700ZS*, *BnaA08G0145800ZS*, *BnaC01G0061700ZS*, and *BnaC03G0759700ZS*, were upregulated by 2.0, 2.8, 3.0, and 4.9 times in activated axillary buds, respectively. Based on the transcriptional levels of TIFY family genes in different states of axillary buds, rapeseed TIFY family members may be involved in the regulation of branch development by a negative means.

### 2.5. TIFY Family Genes Were Involved in the Response of Jasmonic Acid Signaling

**Figure 7:**
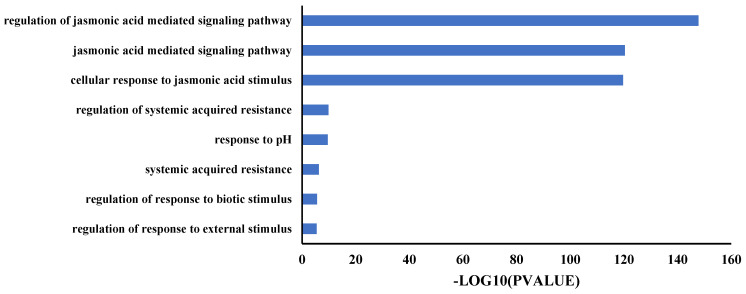
GO enrichment analysis of TIFY family genes. The relevant raw data are listed in Table S3.

Gene ontology (GO) enrichment analysis was carried out to explore the potential biological roles of rapeseed TIFY family genes. The GO analysis was performed on a professional website (https://yanglab.hzau.edu.cn/BnIR/GO, accessed on 7 May 2023) and visualized using GraphPad Prism 9.0 (GraphPad Software, San Diego, CA, USA). The results show that TIFY family genes are mainly involved in three biological processes: regulation of JA-mediated signaling pathway, JA-mediated signaling pathway, and cellular response to JA stimulus. A few TIFY genes are involved in the regulation of systemic acquired resistance and other biological processes (Figure 7).

**Figure 8:**
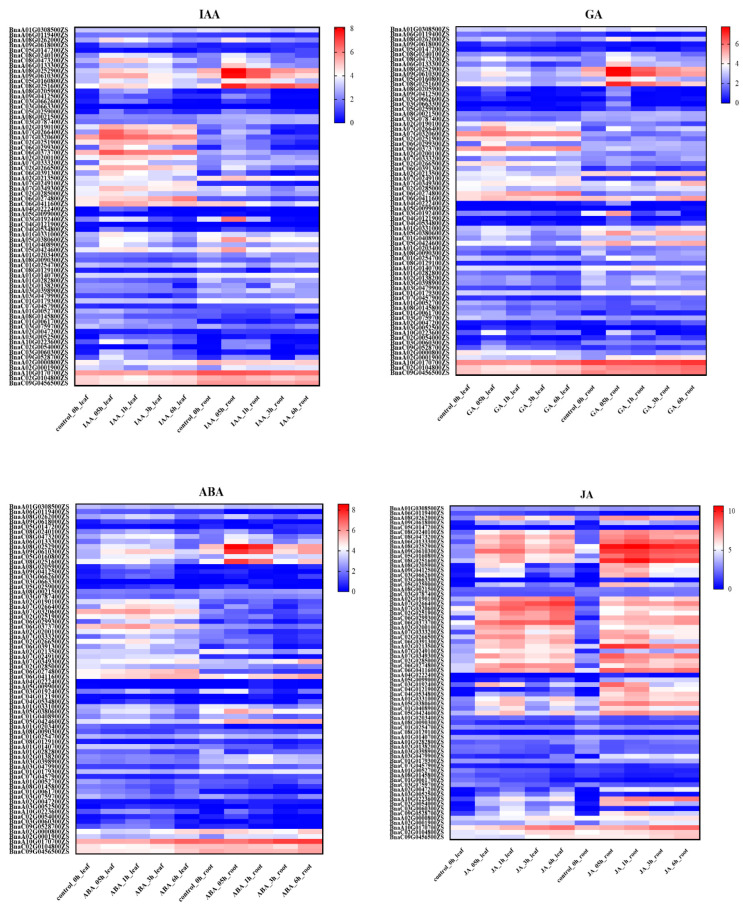
Expression level of TIFY family genes after IAA, GA, ABA, and JA treatments at different times. The heatmap was generated using log2 expression levels (TPM + 1). The bars indicate the log2 expression levels (TPM + 1). The detailed expression values (TPM) of all TIFY genes in leaves and roots are listed in Table S4.

To obtain further insight into the potential roles of TIFY family genes in hormone-mediated biological processes, the expression levels of TIFY family genes were investigated after leaves and roots were treated with JA, 3-Indoleacetic acid (IAA), gibberellin (GA), and abscisic acid (ABA) at four time points (0.5 h, 1 h, 3 h, and 6 h) in ZS11 based on a publicly available RNA-seq database (https://yanglab.hzau.edu.cn/BnIR/expression_zs11, accessed on 9 May 2023). The TIFY family genes displayed distinct expression patterns after ZS11 leaves and roots were treated with JA, IAA, GA, and ABA (Figure 8). Most of the TIFY family genes are strongly induced by JA in leaves and roots, which is consistent with the results of GO enrichment analysis. In addition, most of the genes in the TIFY family that are rapidly upregulated after JA treatment belong to the JAZ subfamily. Previous studies showed that JAZ genes can rapidly respond to JA in maize [38]. Among the JAZ subfamily genes, *BnaA07G0266400ZS* and *BnaC06G0299300ZS* display prominent changes in expression levels. The expression of *BnaA07G0266400ZS* was upregulated by 3.9- and 7.1-fold 6 h after JA treatment in leaves and roots, respectively. The expression of *BnaA10G0223600ZS* was upregulated by 5.5- and 14.0-fold after JA treatment of 6 h in leaves and roots, respectively. The expression patterns of most JAZ subfamily genes are similar in leaves and roots after treatment with JA. The expression of JAZ subfamily genes induced by JA is generally upregulated 0.5 h after treatment and subsequently maintains a high transcriptional level. However, the expression of *BnaC03G0662600ZS* showed the highest transcriptional level at 0.5 h (5.48), rapidly decreased at 3 h (0.82), and increased again at 6 h (2.46) in leaves after JA treatment. Moreover, some JAZ subfamily genes, such as *BnaA02G0000800ZS*, *BnaA02G0001900ZS*, and *BnaC09G0456500ZS,* display a low degree of expression fluctuation after JA treatment. In addition, the expression of *BnaA06G0119400ZS* is slightly downregulated after JA treatment. In general, the JAZ subfamily genes are more rapidly induced by JA.

As shown in Figure 8, a few TIFY family genes are only slightly induced by IAA, GA, and ABA treatments. *BnaA08G0252900ZS*, *BnaA09G0610300ZS*, and *BnaC08G0251600ZS* rapidly respond to IAA, GA, and ABA signaling in roots after treatments. The expression levels of *BnaC06G0299300ZS*, *BnaA07G0333200ZS*, and *BnaC06G0391300ZS* were upregulated by approximately 2-fold at 0.5 h and subsequently gradually returned to normal levels 6 h after IAA, GA, and ABA treatments in leaves. Moreover, IAA seems to induce higher expression of certain TIFY genes than GA and ABA. These results demonstrate that TIFY family members possess distinct roles in hormone-mediated biological processes.

**Figure 9:**
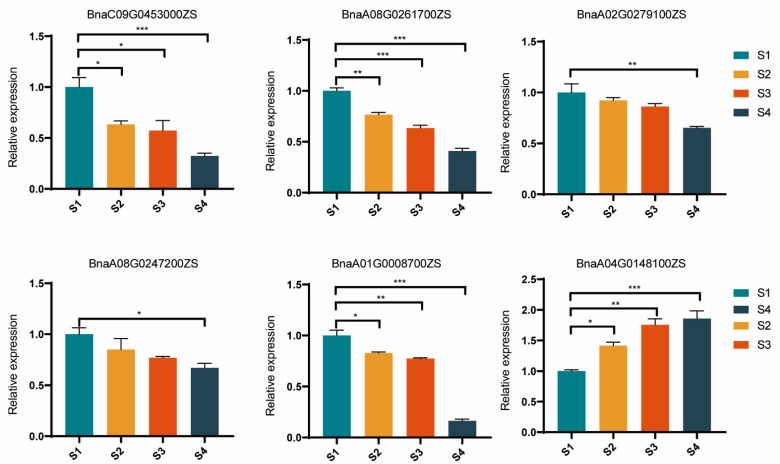
The relative expression of plant hormone-related genes in axillary buds of different states. *BnaC09G0453000ZS* codes cytokinin 7 (*CKX7*); *BnaA08G0261700ZS*, *BnaA02G0279100ZS*, and *BnaA08G0247200ZS* are involved in JA synthesis pathways; *BnaA01G0008700ZS* and *BnaA04G0148100ZS* are involved in auxin metabolic pathways. The expression levels of these genes were calculated with the following equation: 2^−ΔΔCT^. Mean ± standard error is plotted. Significant differences (*p*  < 0.05) are indicated by asterisks. * Represents *p* < 0.05, ** represents *p* < 0.01, *** represents *p* < 0.001.

In addition, the expression levels of key genes that are involved in plant hormone metabolic pathways were investigated in axillary buds. The results are shown in Figure 9. In rapeseed, *BnaA08G0261700ZS*, *BnaA02G0279100ZS*, and *BnaA08G0247200ZS* encode *LOX3*, *AOS*, and *OPCL* enzymes, respectively. *LOX3*, *AOS*, and *OPCL* are primary enzymes in JA biosynthesis [23,39,40]. With the elongation of axillary buds, the expression levels of these genes, which are involved in JA synthesis, decrease gradually and become the lowest in elongated axillary buds (S4). These results imply that JA may play negative roles in branch development. *BnaC09G0453000ZS* encodes cytokinin oxidase/dehydrogenase (*CKX7*), which catalyzes the degradation of cytokinin [41]. In the investigated axillary buds, the expression level of *BnaC09G0453000ZS* was the highest in dormant axillary buds (S1) and decreased to be the lowest in elongating axillary buds (S4), suggesting that *CKX7* may negatively regulate branch development. The tryptophan (Trp)-independent and Trp-dependent pathways are two major pathways of IAA synthesis in plants [42,43]. *BnaA01G0008700ZS* encodes a cytochrome P450 enzyme (*CYP79B2*) that converts tryptophan (Trp) to indole-3-acetaldoxime (IAOX). IAOX is a precursor to IAA and indole glucosinolates [44]. The CYP79B gene family has only been found in *Arabidopsis*, *brassica napus*, and *Sinapis alba* [45,46,47]. The expression level of *BnaA01G0008700ZS* (*CYP79B2*) is the highest in dormant axillary buds (S1) and the lowest in elongating axillary buds (S4). *BnaA04G0148100ZS* (*IAA8*), encoding a transcriptional repressor of auxin response [48], shows the opposite expression pattern to *BnaA01G0008700ZS*. These results suggest that auxin regulates branch development in a negative manner.

## 3. Discussion

The TIFY gene family has been identified in a series of plant species, such as rice [49], *populus trichocarpa* [50], cotton [51], soybean [52], maize [38], wheat [53], and walnut [54]. Here, we identified the TIFY gene family members in *Brassica napus* (ZS11, a representative semi-winter rapeseed variety) and concluded that they may negatively regulate shoot branching by being involved in the activation of axillary buds.

Seventy TIFY family members were identified in rapeseed and were divided into four subfamilies (named JAZ, TIFY, PPD, and ZML subfamilies). Ninety-two TIFY family members were identified in wheat [53]; 24 and 20 TIFY family members were identified in *populus trichocarpa* [50] and rice [49], respectively. Differences in the number of TIFY family members may be related to the size of the genomes of different species. The tissue expression profile of TIFY family genes showed that these genes were highly expressed in the stem, mature leaf (leaf 23), 4 mm flower bud, and silique (30 days after flowering). Most of the TIFY genes showed high transcript levels in the leaves of rice [49] and cotton [51]. The TIFY family genes of *populus trichocarpa* [50] had abundant expression levels in the phloem, xylem, and mature leaves. TIFY family genes were also expressed in the stems and leaves of maize [38]. Generally, the majority of TIFY genes are expressed in different tissues. Most of the JAZ subfamily genes show higher expression levels than genes of other subfamilies (TIFY, PPD, and ZML). The diverse expression patterns of TIFY family genes indicated that they must be involved in multiple biological processes and play important roles in plants. Of the 13 JAZ genes in *Arabidopsis thaliana*, 10 promote plant growth and reproductive adaptation [55]. In rice, overexpression of *OsTIFY11a* enhances tolerance to drought and salt, and the seeds of overexpressed plants have significantly higher germination rates than the non-transgenic seeds under drought and salt stresses [17]. ZIM subfamily genes can lead to the elongation of hypocotyls and petioles of *Arabidopsis thaliana* [1]. These studies suggested the functional diversity of TIFY family genes. However, the roles and mechanisms of growth and development regulated by TIFY family genes are unknown. Therefore, more functions of TIFY family genes need to be explored in the future.

At present, multiple studies have found that JA is involved in the regulation of seed and spike development, which are crucial to improving yield. For example, overexpression of Arabidopsis *JMT*, encoding the jasmonate carboxy methyl transferase and converting jasmonate to methyl jasmonate (MeJA), leads to a decrease in spike number and filling rate per spike, resulting in decreased grain yield in rice. This low yield is due to the abnormal spikelet development caused by a 6-fold increase in MeJA content in young ears [56]. *NOG1* (NUMBER GRAINS 1) also affects grain yield by affecting JA biosynthesis. Its high expression leads to the downregulation of genes associated with the JA biosynthetic pathway, which results in reduced endogenous JA levels. These lead to an increase in the number of grains per ear and per plant in rice [57]. JA signaling affects spikelet development by regulating floral organ identity and floral meristem determinacy in rice [58]. In addition, spikelet development is regulated by another gene, *OsPEX5*, which can promote JA biosynthesis. Its loss of function causes spikelet morphology abnormalities, such as excess glume, abnormal lemma and glume, the formation of side flowers, and distorted numbers of stamens and pistils [59]. JA signaling negatively regulates integument cell proliferation and represses seed sizes in *Arabidopsis thaliana* [60]. Our results are similar to the above conclusions; the expression of these genes which are involved in the synthesis of JA decreases gradually with the development of axillary buds and shows the lowest level in elongated axillary buds (S4). These results suggest that JA may play negative roles in branch development.

After JA treatment, the transcription of the JAZ subfamily members in rapeseed was induced in leaves [61]. Most of the TIFY genes in wheat were highly expressed after JA treatment [53]. The expression of most of the JAZ genes in cotton rapidly increased at first and then decreased to normal levels after exogenous JA or MeJA treatments [51]. Most of the maize JAZ subfamily genes were found to be JA-inducible. The expression levels of *ZmJAZ8*, *11*, *12*, *18*, *25*, *31*, *32*, and *33* were upregulated at 6 h after JA treatment [38]. The results of GO enrichment analysis and exogenous hormone treatments showed that JAZ subfamily genes in rapeseed strongly responded to JA, implying the JAZ subfamily genes played much more important roles in JA-mediated branch development.

The functions of TIFY family genes in shoot branching are unknown; we performed transcriptomic sequencing of axillary buds (early stage of branching) in different development states. The transcripts of TIFY family genes highly accumulate in dormant axillary buds and significantly decrease in activated axillary buds. Most of the genes significantly decrease at the transcriptional level on activation of axillary buds, which implies that TIFY family genes may be involved in the regulation of branch development. It has been reported that JA negatively regulates the branch growth of pear trees and JA downstream regulatory genes play crucial roles in the regulation of branching [62]. Rapeseed is an important oil crop, and the number of branches determines the number of siliques and ultimately determines yield. Increasing the number of branches in rapeseed can improve canopy structure and further increase yield. The TIFY family genes have been reported to be involved in the response to *Sclerotinia sclerotiorum*, freezing, salicylic acid (SA), methyl jasmonate (MeJA), PEG, NaCl [57], and heavy metal stress [63] in rapeseed. Here, we present the novel roles of TIFY family genes in negatively regulating the development of branching in the early stage. Therefore, it is meaningful to dissect the roles of TIFY family genes in branch development. The new insights in our study may be applicable in shaping plant architecture and further improving yield in rapeseed.

## 4. Materials and Methods

### 4.1. Plant Materials, Sampling, RNA Extraction, and RNA-Seq

ZS11 was from the Oil Crops Research Institute of the Chinese Academy of Agricultural Sciences (CAAS). ZS11 plants were grown under conventional field management and growth conditions at Yangluo Experimental Field (N:30°42′35.78″, E:114°30′49.05″), Oil Crops Research Institute of Chinese Academy of Agricultural Sciences, Wuhan. Axillary buds of different developmental states were collected from the bottom, lower, middle, and upper parts of the plant during the bolting period and named S1 (dormant axillary buds), S2 (temporarily axillary buds), S3 (being activated axillary buds), and S4 (already activated and elongated axillary buds), respectively. Samples were immediately frozen in liquid nitrogen and stored at −80 ℃ in a freezer for RNA-seq; there were three biological replicates for each type of axillary bud sample, with three plants in each replicate.

Total RNA extraction followed the instructions of the manufacturer of Plant RNA Kit (R6827-01, Omega, GA, USA). cDNA libraries were constructed using Poly-A Purification TruSeq library reagents and sequenced on an Illumina platform. The clean reads were mapped to the rapeseed (ZS11.v0) reference genome (https://yanglab.hzau.edu.cn/BnIR/genome_data, accessed on 17 November 2022) [32] using TopHat2 software (http://ccb.jhu.edu/software/tophat, accessed on 23 November 2022) [64]. The read counts of each gene were calculated using HTSeq 2.0 software (Python Software Foundation, VA, USA) [65]. Differentially expressed genes (DEGs) between two sample groups were analyzed using the DESeq R package. False discovery rate (FDR) <0.01 and fold changes (FC) ≥2 were set as the thresholds for significant DEGs. GO enrichment analysis of the DEGs was performed using the GOseq R package.

### 4.2. Identification of TIFY Gene Family in Brassica napus

All the members of the TIFY gene family were identified in the ZS11.v0 genome (https://yanglab.hzau.edu.cn/BnIR/genome_data, accessed on 3 April 2023) [32]. BlastP and HMMER (HMMER3.0 package) searching were carried out using all the amino acid sequences of *Arabidopsis* TIFY proteins as queries to search for all TIFY members in the ZS11.v0 genome. Then, the redundant genes were eliminated from the HMMER and BLASTP outcomes. The *Ensembl genomes* [66] were used to predict the isoelectric point, subcellular localization, and molecular weight of the TIFY genes.

### 4.3. Sequence Alignments and Phylogenetic Analyses

The phylogenetic analysis of TIFY members in *Brassica napus* and *Arabidopsis thaliana* was performed using MEGA X (Mega Limited, Auckland, New Zealand) [33]. The TIFY family gene sequence alignment and phylogenetic tree were generated using MEGA X based on the neighbor-joining method with 1000 bootstrap replicates. The final circular phylogenetic tree was visualized using Evolview (https://evolgenius.info/, accessed on 6 April 2023) [67].

### 4.4. Chromosomal Location

The location information of TIFY genes on the chromosomes was retrieved from the ZS11.v0 genome and visualized using Gene Location Visualize from the GTF/GFF function module of TBtools II (TBtools software, Guangzhou, China) [68].

### 4.5. Exon/Intron Structure Analyses and Conserved Motif Identification

The genomic sequences and coding sequences (CDSs)of rapeseed TIFY family genes were extracted from the ZS11.v0 genome database and compared in gene structure display server programs to determine the exon/intron compositions of TIFY genes. GSDS [69] (http://gsds.cbi.pku.edu.cn/, accessed on 17 April 2023) was used to visualize the exon–intron structures of rapeseed TIFY genes. In genetics, sequence motifs are special, identifiable nucleotide or amino acid sequences. The online version of MEME [70] (http://meme.ncbr.net/meme/cgi-bin/meme.cgi, accessed on 20 April 2023) was used for motif structure prediction. The predicted number of motifs was 10, the best match length was 6–50, and the other parameters were default values.

### 4.6. Expression Analyses and Heatmap Analyses

The analysis of the expression of TIFY family genes on dormant (S1), temporarily dormant (S2), being activated (S3), and elongating (S4, already activated) axillary buds was based on our RNA-seq (unpublished data). The data of the tissue expression profile of TIFY genes were obtained from the public database website of the ZS11 transcriptome information resource (http://yanglab.hzau.edu.cn/BnTIR/, accessed on 27 April 2023). The relative expression levels of TIFY family genes after treatments with JA, IAA, GA, and ABA were based on a publicly available database (http://yanglab.hzau.edu.cn/BnTIR/, accessed on 9 May 2023). All heatmaps were drawn using GraphPad Prism 9.0 software (GraphPad Software, San Diego, CA, USA).

### 4.7. Gene Ontology (GO) Enrichment Analysis

In order to identify the enrichment pathways of TIFY family genes, GO analysis was performed on all the identified TIFY genes. The GO analysis of TIFY genes was performed on a professional website (https://yanglab.hzau.edu.cn/BnIR/GO, accessed on 7 May 2023) and visualized with GraphPad Prism 9.0 software (GraphPad Software, San Diego, CA, USA).

### 4.8. qRT-PCR Analysis

To investigate the expression of TIFY genes in axillary buds, RNA was extracted from the axillary buds of ZS11 plants in the bolting period (same as Section 4.1). Coding DNA (cDNA) was synthesized via reverse transcription reaction using the PrimeScript™ RT reagent Kit (RR047A, Takara, Kyoto, Japan). Real-time quantitative PCR was performed according to the instructions of the manufacturer of SYBR Green PCR master mix (71782420, LightCycler^®^ 480 SYBR^®^ Green 1 Master, Roche, Basel, Switzerland) on a Roche LightCycler^®^ 96 instrument (Roche, Basel, Switzerland). The PCR reaction volume comprised 5 μL of SYBR Green I Master, 1 μL of cDNA (10 ng), 1 μL of primer mix of 10 μM, and 3 μL of DNase/RNase-free water. All primers are shown in Supplemental Table S5. The expression of *Bn actin 7* was used as the reference. The expression levels of genes were calculated with the equation 2^−ΔΔCT^ and visualized with GraphPad Prism 9.0 software (GraphPad Software, San Diego, CA, USA). SPSS 18.0 was used for statistical analysis (Variance treatment, Duncan’s test), and significance was assessed based on a one-way ANOVA test.

## 5. Conclusions

TIFY family genes play important roles in plant growth and development, but their functions in shoot branching are unknown. In this project, a total of 70 TIFY members were identified in rapeseed and divided into four subfamilies. TIFY family genes were highly expressed in stems, flower buds, and siliques. TIFY family genes strongly responded to jasmonic acid signaling and the jasmonic acid pathway based on GO enrichment analysis and hormone treatment analyses. To explore the potential roles of TIFY family genes in shoot branching, the expression levels of TIFY family genes in four sequential developing stages of axillary buds (prerequisites for shoot branching) were detected. The transcripts of TIFY family genes highly accumulated in dormant axillary buds and significantly decreased in activated axillary buds. Here, we present the novel insight that TIFY family genes may be involved in the negative regulation of jasmonic acid-mediated branch development.

## Figures and Tables

**Table 1:** Lists and basic characteristics of the rapeseed TIFY family genes.

Gene Name	Chromosome Location	Direction	AtGI	At Name	Protein Length (aa)	Molecular Weight (kDa)	Isoelectric Point (pI)	Subcellular Prediction
*BnaA01G0052700ZS*	A01:2859984…2861895	+	AT4G32570	TIFY8	310	33.56	6.51	nucl
*BnaA01G0140700ZS*	A01:8392342…8394246	+	AT4G24470	GATA25	320	35.09	9.49	nucl
*BnaA01G0203400ZS*	A01:12583796…12585951	+	AT4G14720	TIFY4B	130	13.73	4.29	nucl
*BnaA01G0282800ZS*	A01:26329701…26330093	+	AT4G24470	GATA25	367	40.68	7.35	chlo
*BnaA01G0308500ZS*	A01:28824803…28828698	−	AT3G21175	GATA24	353	37.58	9.5	nucl
*BnaA01G0331000ZS*	A01:30506077…30510006	−	AT3G17860	TIFY6B	345	37.44	10.12	nucl
*BnaA02G0000800ZS*	A02:293262…294602	−	AT5G20900	TIFY3B	216	22.47	4.7	cyto
*BnaA02G0001900ZS*	A02:350665…352002	+	AT5G20900	TIFY3B	112	11.96	4.35	cysk
*BnaA02G0047200ZS*	A02:2647926…2649211	+	AT5G13220	TIFY9	274	29.34	10.43	nucl
*BnaA02G0138200ZS*	A02:7624707…7625045	−	AT4G24470	GATA25	273	30.16	9.64	nucl
*BnaA02G0190100ZS*	A02:11734586…11736653	+	AT1G70700	TIFY7	240	26.20	9.6	nucl
*BnaA02G0200100ZS*	A02:12545103…12546473	−	AT1G72450	TIFY11B	177	19.36	10.52	cyto
*BnaA02G0213500ZS*	A02:13411661…13412862	−	AT1G74950	TIFY10B	212	23.99	10.64	golg
*BnaA03G0052500ZS*	A03:2524449…2525938	+	AT5G13220	TIFY9	112	11.96	4.35	cysk
*BnaA03G0398900ZS*	A03:21529159…21529497	−	AT4G24470	GATA25	261	28.28	6.41	nucl
*BnaA03G0479900ZS*	A03:26610896…26612549	+	AT4G24470	GATA25	223	25.25	10.44	chlo
*BnaA04G0222400ZS*	A04:21368091…21368591	+	AT2G34600	TIFY 5B	119	13.70	10.24	nucl
*BnaA05G0099000ZS*	A05:5800314…5800869	−	AT2G34600	TIFY 5B	116	13.06	8.89	nucl
*BnaA05G0380600ZS*	A05:37865369…37867939	−	AT3G17860	TIFY6B	336	35.87	9.9	nucl
*BnaA06G0119400ZS*	A06:6986490…6987776	−	AT1G17380	TIFY11A	266	29.80	9.7	nucl
*BnaA06G0133300ZS*	A06:7845736…7847029	+	AT1G19180	TIFY10A	254	27.30	10.51	chlo
*BnaA07G0249100ZS*	A07:24141740…24144675	+	AT1G74950	TIFY10B	224	24.59	9.56	nucl
*BnaA07G0266400ZS*	A07:25167684…25168787	−	AT1G70700	TIFY7	212	23.41	9.12	mito
*BnaA07G0320600ZS*	A07:28617818…28620360	+	AT1G70700	TIFY7	266	28.64	10.45	nucl
*BnaA07G0333200ZS*	A07:29319157…29320650	−	AT1G72450	TIFY11B	274	30.60	9.62	nucl
*BnaA07G0349300ZS*	A07:30135515…30136690	−	AT1G74950	TIFY10B	245	26.76	9.62	nucl
*BnaA08G0021500ZS*	A08:1797523…1798971	−	AT1G51600	GATA28	318	35.01	9.58	nucl
*BnaA08G0090300ZS*	A08:15229435…15231385	−	AT4G14720	TIFY4B	357	38.75	9.11	nucl
*BnaA08G0145800ZS*	A08:19339471…19341512	+	AT4G32570	TIFY8	130	14.97	10.39	nucl
*BnaA08G0205900ZS*	A08:22909636…22910221	−	AT1G30135	TIFY5A	311	33.96	6.45	nucl
*BnaA08G0252900ZS*	A08:25292226…25293349	−	AT1G19180	TIFY10A	259	28.31	9.99	nucl
*BnaA08G0262000ZS*	A08:25690409…25691687	+	AT1G17380	TIFY11A	267	29.71	9.57	nucl
*BnaA09G0412500ZS*	A09:47302666…47303247	−	AT1G30135	TIFY5A	133	15.23	9.83	nucl
*BnaA09G0610300ZS*	A09:60116951…60119612	−	AT1G19180	TIFY10A	273	29.82	10	nucl
*BnaA09G0618000ZS*	A09:60520671…60529802	+	AT1G17380	TIFY11A	315	35.08	10.34	chlo
*BnaA10G0170700ZS*	A10:20196301…20197382	−	AT5G20900	TIFY3B	203	21.43	7.58	nucl
*BnaA10G0223600ZS*	A10:22916557…22919394	−	AT5G13220	TIFY9	196	21.82	10.65	nucl
*BnaC01G0061700ZS*	C01:3490282…3492250	+	AT4G32570	TIFY8	304	33.06	6.64	nucl
*BnaC01G0179300ZS*	C01:13276298…13278294	+	AT4G24470	GATA25	314	34.53	8.44	nucl
*BnaC01G0254700ZS*	C01:20032455…20034770	−	AT4G14720	TIFY4B	353	37.52	9.38	nucl
*BnaC01G0408900ZS*	C01:47295575…47298846	−	AT3G17860	TIFY6B	344	37.27	10.12	nucl
*BnaC02G0054000ZS*	C02:3401968…3404083	+	AT5G13220	TIFY9	221	23.08	4.72	nucl
*BnaC02G0104800ZS*	C02:6810525…6811864	+	AT5G20900	TIFY3B	274	29.41	10.51	cyto
*BnaC02G0251900ZS*	C02:23433804…23435871	+	AT1G70700	TIFY7	272	29.95	7.71	nucl
*BnaC02G0266500ZS*	C02:25372570…25373915	−	AT1G72450	TIFY11B	240	26.20	9.75	nucl
*BnaC02G0285000ZS*	C02:27147851…27149030	−	AT1G74950	TIFY10B	196	21.80	10.68	nucl
*BnaC03G0060300ZS*	C03:3153036…3154515	+	AT5G13220	TIFY9	113	13.03	8.78	chlo
*BnaC03G0192400ZS*	C03:11016512…11016853	+	AT2G34600	TIFY 5B	223	25.20	10.44	nucl
*BnaC03G0662600ZS*	C03:64076368…64076942	+	AT1G30135	TIFY5A	130	14.97	10.39	nucl
*BnaC03G0663300ZS*	C03:64131128…64131740	+	AT1G30135	TIFY5A	144	16.74	8.64	nucl
*BnaC03G0759700ZS*	C03:73567925…73569989	+	AT4G32570	TIFY8	358	38.78	9.17	nucl
*BnaC03G0787400ZS*	C03:76176548…76178066	+	AT1G51600	GATA28	296	32.35	6.18	nucl
*BnaC04G0121900ZS*	C04:11013009…11013565	−	AT2G34600	TIFY 5B	116	13.13	9.33	nucl
*BnaC04G0534800ZS*	C04:65634871…65635324	+	AT2G34600	TIFY 5B	121	13.65	9.26	nucl
*BnaC05G0147200ZS*	C05:9303032…9304540	−	AT1G17380	TIFY11A	256	28.40	10.36	nucl
*BnaC05G0160800ZS*	C05:10437306…10438599	+	AT1G19180	TIFY10A	254	27.26	10.3	nucl
*BnaC05G0259000ZS*	C05:21020991…21021569	+	AT1G30135	TIFY5A	132	15.24	9.83	nucl
*BnaC05G0424600ZS*	C05:47654460…47657298	−	AT3G17860	TIFY6B	335	35.76	9.85	nucl
*BnaC06G0274800ZS*	C06:38103129…38104304	+	AT1G74950	TIFY10B	224	24.46	9.77	nucl
*BnaC06G0299300ZS*	C06:40408436…40409553	−	AT1G70700	TIFY7	212	23.32	9.12	nucl
*BnaC06G0373700ZS*	C06:47272615…47275070	+	AT1G70700	TIFY7	266	28.53	10.12	nucl
*BnaC06G0391300ZS*	C06:48490374…48491996	−	AT1G72450	TIFY11B	271	30.32	10.11	nucl
*BnaC06G0411600ZS*	C06:49662272…49663471	−	AT1G74950	TIFY10B	246	27.24	9.17	nucl
*BnaC07G0457900ZS*	C07:55670963…55672463	+	AT4G24470	GATA25	259	28.08	6.23	nucl
*BnaC08G0129100ZS*	C08:23055859…23058016	−	AT4G14720	TIFY4B	362	39.79	8.43	ER
*BnaC08G0240100ZS*	C08:33426315…33427601	−	AT1G17380	TIFY11A	268	29.79	9.1	nucl
*BnaC08G0251600ZS*	C08:34242963…34244270	+	AT1G19180	TIFY10A	264	28.74	10.22	nucl
*BnaC08G0473200ZS*	C08:50094384…50095986	+	AT1G17380	TIFY11A	343	38.00	10.11	nucl
*BnaC09G0456500ZS*	C09:56962395…56963463	−	AT5G20900	TIFY3B	201	21.25	6.95	cyto
*BnaC09G0528700ZS*	C09:62610296…62611889	−	AT5G13220	TIFY9	199	22.46	10.73	nucl

nucl, chlo, cyto, cysk, golg, mito, and ER indicate nucleus, chloroplast, cytoplasm, cytoskeleton, Golgi apparatus, mitochondrion, and endoplasmic reticulum, respectively.

## Data Availability

Data are contained within the article and its Supplementary Files.

## Supplementary Materials

The following supporting information can be downloaded at: https://www.mdpi.com/article/10.3390/ijms242317114/s1.
